# Influence of body variables in the development of metabolic syndrome—A long term follow-up study

**DOI:** 10.1371/journal.pone.0192751

**Published:** 2018-02-12

**Authors:** Chiara Pavanello, Anna Maria Zanaboni, Sabrina Gaito, Margherita Botta, Giuliana Mombelli, Cesare R. Sirtori, Massimiliano Ruscica

**Affiliations:** 1 Centro E. Grossi Paoletti, Dipartimento di Scienze Farmacologiche e Biomolecolari, Università degli Studi di Milano, Milan, Italy; 2 Dipartimento di Informatica, Università degli Studi di Milano, Milan, Italy; 3 Dipartimento di Scienze Farmacologiche e Biomolecolari, Università degli Studi di Milano, Milan, Italy; 4 Dyslipidemia Center, A.S.S.T. Grande Ospedale Metropolitano Niguarda, Milan, Italy; Beijing Key Laboratory of Diabetes Prevention and Research, CHINA

## Abstract

**Objectives:**

The body variable associated with the diagnosis of Metabolic Syndrome (MetS) is an elevated waist circumference (WC), although a number of other variables have been suggested. Among these, an elevated waist-to-height ratio (WHtR), *ie* a value higher than 0.5, that may identify abnormality, independently from height. An elevated WHtR provided the best correlation with MetS in a prior study in a large Italian population. In order to assess the validity of this conclusion, a long-term follow-up study re-examined this population, also in order to detect possible associations with cardiovascular (CV) risk.

**Methods and results:**

1,071 subjects with a complete follow-up of over 6 years were evaluated with a comparative assessment of the three anthropometric variables, namely WHtR, WC and body mass index (BMI). WHtR≥ 0.5 had the highest sensitivity for the identification of MetS, both in males and females (94.1% and 86.7% respectively). WHtR was of reduced specificity, occurring, yet less frequently (17.7% in males and 30% in females), in patients without MetS. By contrast, enlarged WC occurred with a lower frequency in male patients who developed MetS (30.2%) whereas in females, frequency was higher than in males (69.3%). Finally, a BMI≥ 25 kg/m^2^ had intermediate sensitivity and specificity regardless of gender. WC showed the highest odds ratio (2.62, 95%CI: 1.18–5.78) for the prediction of CV occurrence.

**Conclusion:**

The present study confirms WHtR as an excellent screening tool in identifying MetS carriers, but, different from reports in other countries, it shows a lower specificity in our population.

## 1. Introduction

The profile of Metabolic Syndrome (MetS), *ie* the association of biochemical features (low high-density lipoprotein (HDL) cholesterol, hypertriglyceridemia and hyperglycemia), body variable (i.e. waist circumference (WC) ≥ 102 cm in male and ≥ 88 cm in female) and elevated blood pressure (BP), is widely accepted. There is, however, some variability in the diagnostic criteria, particularly relative to the body variable/s [1]. Further, the assessment of the relative influence of each risk factor on the cardiovascular (CV) risk has led to partially discordant conclusions [2]. Indeed, hypertension is likely to be the most significant MetS variable associated with arterial disease [3]; while other authors favor low HDL-C [4], and some others abdominal obesity [5] or hyperglycemia [6].

The recommendation to measure WC rather than body mass index (BMI) has been based on the central role of abdominal obesity in MetS [7], but the use of WC or, alternatively, of BMI as a predictor of CV risk is still debated, since contradictory data have come out of epidemiological studies [8, 9]. Among others, the Reaven's group has repeatedly indicated that body variables do not significantly contribute to the CV risk, or even to the diagnosis of MetS [10]. The weakness of WC as a risk index is the considerable variability in threshold [1], among others indicating that there may be little difference in the predictive value between larger or smaller thresholds (Europid or American) [1]. Recently, however, a Swedish study indicated a higher CV risk for individuals with a larger WC, compared to those with a smaller one [11]. Thus, it was of special interest to evaluate a large series of well-characterized individuals from the Mediterranean area for whom an early report indicated that the waist-to-height ratio (WHtR) was the best predictor of MetS (more so than WC) [12] and, further, that raised WHtR was associated with an increased progression of carotid intima-media thickness (cIMT) [13].

The follow-up of this patient series has given the opportunity to re-evaluate the reliability of these findings after a period of 6 years. Hence, this study attempted to either confirm or refute the predictive value of WHtR, compared to WC and BMI, on the occurrence of MetS. The possible association of these variables with the CV risk was also evaluated.

## 2. Methods

### 2.1 Subjects

The baseline study population has been previously described. Briefly, 1,104 subjects were recruited among those attending the Dyslipidemia Center of the ASST Grande Ospedale Metropolitano Niguarda, Milan in 2008 [12]. After 6 years of follow-up the cohort was composed of 1,071 subjects, *ie* 539 males (57.8 ± 12.8 years (y), mean ± standard deviation (SD) and 532 females (63.9 ± 11.1 y, mean ± SD). The study protocol of this observational study was approved by the Ethical Committee of the ASST Grande Ospedale Metropolitano Niguarda. A signed consent form was obtained from all of the participants. The study conforms to the Ethical guidelines of the 1975 Declaration of Helsinki. Vascular events at baseline and over a period of 6 years were recorded in the medical electronic records by a hospital physician. Pre-existing CV disease was defined in participants with a previous diagnosis of angina, acute myocardial infarction, stroke and peripheral arterial disease. All determinations were performed in the morning of the clinical examination.

Blood samples were taken after a 12 h overnight fast. Serum lipids, lipoproteins and glucose were determined by standard procedures as used in the institution [14, 15]. Weight and height were measured using the same equipment and WC was measured at umbilical level [16] on subjects standing and breathing normally as indicated by Kagawa *et al*. [16] These Authors compared different “waist” measurements, calculated using the narrowest point between the lower costal border and the top of the iliac crest and at the level of the umbilicus and concluded that sensitivity of measurement of excess percent body fat and percent total fat increased more significantly by using the umbilical measurement, particularly in females. Mason and Katzmarzyk [17] further indicated that the prevalence of MetS is modestly influenced by the anatomic site of WC measurement. WHtR was defined as the ratio between WC and height. BMI was calculated as weight in kilograms divided by square of the height in meters. Systolic and diastolic BPs were measured in subjects in a sitting position with a mercury sphygmomanometer after a 2-minute rest.

### 2.2 Definition of MetS

To allow a direct comparison with previous data, MetS was diagnosed according to the American Heart Association/National Heart, Lung and Blood Institute (AHA/NHLBI) guidelines [18, 19] as the clustering of three or more of the following features, namely, systolic BP (SBP) ≥ 130 mmHg and/or diastolic BP (DBP) ≥ 85 mmHg and/or currently receiving medications; fasting glucose (FG) ≥ 100 mg/dL and/or currently receiving medication; fasting triglycerides (TG) ≥ 150 mg/dL and/or currently use of medications; HDL cholesterol < 40 mg/dL for males and < 50 mg/dL for females and WC ≥ 102 cm for males and ≥ 88 cm for females.

### 2.3. Statistical analysis

Continuous variables are presented as means ± SD, and categorical variables as cases (n) or percentages (%). Prevalence of obesity-related risk factors in MetS are presented as percentage values and stratified by gender. Sensitivity and specificity for the identification of MetS, as the clustering of two or more coronary risk factors plus the anthropometric one, were investigated using each of the proposed indices, that is, an enlarged WC according to AHA/NHLBI criteria, BMI ≥ 25 kg/m^2^ and WHtR ≥ 0.5.

Comparisons between baseline and follow-up were performed using paired Student’s 2-tailed t-test. Receiver operating characteristic (ROC) curves for the clustering of two or more coronary risk factors were produced for each anthropometric variable; the values of the indices that maximized the Youden index (sensitivity + specificity– 1) were defined as optimal. Odds ratios and their 95% confidence intervals (CI), were calculated by univariate logistic regression analysis. All reported P values < 0.05 were considered as statistically significant. Statistical analyses were carried out using the SPSS statistical package (version 19.0; SPSS, Inc., Chicago, IL).

## 3. Results

### 3.1 Patient characteristics

The characteristics of study participants at baseline and after the 6-year follow-up are presented in Table 1. The increase in the use of statins, likely related to the higher number of patients in secondary prevention, was accompanied by the reduction of low-density lipoprotein (LDL) cholesterol and the increase of HDL-cholesterol. Notably, FG and the prevalence of type 2 diabetes mellitus increased at follow-up in both genders, possibly related to an increment in statin usage [20]. Relative to the percentage of diagnostic criteria at follow-up, 20.8% of subjects were carriers of 3, 12.0% of 4 and 3.9% of 5 diagnostic criteria. These percentages were almost identical to those observed at baseline (*ie* 20.8%, 12.4% and 2.8%, respectively). A small subgroup (10.5% of the total sample) had newly diagnosed MetS at the follow-up.

**Table 1:** Clinical and metabolic characteristics of patients included in the 6-year follow-up analyses.

		Males (n = 539)			Females (n = 532)		
		Baseline	Follow-up	P	Baseline	Follow-up	P
Age years	(mean ± SD)	57.8 ± 12.8	63.8 ± 12.8		63.9 ± 11.1	69.9 ± 11.1	
BMI	(kg/m^2^)	26.0 ± 3.0	26.1 ± 3.2	0.017	24.5 ± 3.7	24.6 ± 3.9	0.270
WC	(cm)	94.3 ± 8.1	95.4 ± 8.4	0.000	87.3 ± 10.7	88.2 ± 10.6	0.000
Total cholesterol	(mg/dL)	213.9 ± 44.3	212.2 ± 45.9	0.401	230.7 ± 41.8	229.3 ± 43.2	0.461
LDL cholesterol	(mg/dL)	138.1 ± 47.6	132.0 ± 47.6	0.001	150.5 ± 39.3	141.8 ± 41.0	0.000
TG	(mg/dL)	162.3 ± 161.3	157.8 ± 126.2	0.473	116.3 ± 60.6	121.6 ± 65.6	0.034
HDL cholesterol	(mg/dL)	43.4 ± 11.8	48.6 ± 13.0	0.000	57.0 ± 15.9	63.2 ± 16.2	0.000
FG	(mg/dL)	90.6 ± 16.9	95.5 ± 21.6	0.000	85.3 ± 14.8	89.3 ± 15.7	0.000
DBP	(mmHg)	77.3 ± 8.3	80.0 ± 7.8	0.000	77.3 ± 7.9	79.3 ± 7.2	0.000
SBP	(mmHg)	128.1 ± 13.6	130.5 ± 13.5	0.000	130.5 ± 15.4	131.1 ± 14.0	0.309
T2DM	(%)	51 (9.5)	65 (12.1)	0.003	25 (4.7)	43 (8.0)	0.001
Hypertension	(%)	382 (70.9)	394 (73.1)	0.261	381 (70.6)	397 (73.6)	0.154
Primary prevention	(%)	405 (75.1)	392 (72.7)	0.000	482 (89.5)	469 (87.0)	0.000
Secondary prevention	(%)	134 (24.9)	147 (27.3)	0.000	57 (10.5)	70 (13.0)	0.000
BMI ≥ 25 kg/m^2^	(%)	57.5 (310)	61.1 (329)	0.022	41.2 (219)	42.2 (225)	0.466
WHtR ≥ 0.5	(%)	86.1 (464)	89.1 (480)	0.019	74.8 (398)	77.2 (411)	0.094
WC ≥ 102 cm (males); ≥ 88 cm (females)	(%)	18.4 (99)	21.5 (116)	0.029	47.2 (251)	51.7 (275)	0.001
Statins	(%)	228 (42.3)	258 (47.9)	0.000	273 (50.7)	286 (53.1)	0.002
Fibrates	(%)	123 (22.8)	106 (19.7)	0.001	46 (8.5)	51(9.4)	0.960
Antihypertensives	(%)	247 (45.8)	266 (49.4)	0.000	248 (46.0)	263 (48.8)	0.000

Values are expressed as means ± SD or as number of cases and percentage. Comparisons between baseline and follow-up were calculated by paired t-test. BMI, body mass index; WC, waist circumferences; LDL, low-density lipoprotein; TG, triglycerides; HDL, high-density lipoprotein; FG, fasting glucose; DBP, diastolic blood pressure; SBP, systolic blood pressure; T2DM, type 2 diabetes mellitus; WHtR, waist-to-height ratio.

### 3.2 Prevalence of anthropometric variables and cardio-metabolic risk factors of MetS

The relative proportion of obesity-related risk factors, defined by the three proposed anthropometric indices (BMI ≥ 25 kg/m^2^, WHtR ≥ 0.5 and WC ≥ 88 cm for females and 102 cm for males) remained almost unchanged over the baseline data, with a WHtR ≥ 0.5 being overall more prevalent (89.1% and 77.2%, in males and females, respectively). A prevalence of all the three factors increased significantly from baseline in males, whereas in females only the presence of an enlarged WC was raised at follow-up (from 47.2% to 51.7%). Interestingly, altered anthropometric indices remained higher in males versus females, with the exception of WC, retaining an almost two-fold higher prevalence in females (Table 1).

As a further step, we assessed which obesity-related index was more strongly associated to the clustering of more than one non-anthropometric variable for MetS. Thus, as shown in Table 2, we calculated the prevalence of MetS using the three proposed anthropometric variables as a substitute for the classical anthropometric criterion. WHtR ≥ 0.5 gave the highest prevalence of MetS in males, followed by BMI ≥ 25 kg/m^2^. Conversely, in females, there was little difference in the predictive value among the three body variables.

**Table 2:** Prevalence (%) of metabolic syndrome based on anthropometric variables.

*Presence of obesity-related risk factor based on anthropometric index*		*Prevalence of Metabolic Syndrome (%)*				
	Males (n = 539)			Females (n = 532)		
	Baseline	Follow-up	P value	Baseline	Follow-up	P value
BMI ≥ 25 kg/m^2^	51.9	49.5	0.234	31.8	32.9	0.541
WHtR ≥ 0.5	57.9	55.4	0.234	38.4	39.7	0.522
WC ≥ 102 cm (males); ≥ 88 cm (females)	38.4	38.3	1.000	33.7	35.2	0.438

MetS, metabolic syndrome; BMI, body mass index; WHtR, waist-to-height ratio; WC, waist circumference

### 3.3 Sensitivity and specificity of anthropometric variables on the identification of MetS and association with CV events

Specificity and sensitivity of the proposed anthropometric variables in the identification of the clustering of two or more risk factors of MetS are shown in Table 3. Sensitivity, *ie* the percent association with a correctly identified MetS, was 94.1 in males and 86.7 in females, for a WHtR > 0.5, clearly indicating an optimal association of this variable with a well-defined MetS. WHtR was of reduced specificity; indeed, it occurred, albeit less frequently (17.7% in males and 30% in females) in patients without MetS.

**Table 3:** Sensitivity and specificity for the identification of clustering of two or more risk factors of MetS in both genders.

	**Males** (n = 539)					
	Sensitivity (%)			Specificity (%)		
*Two or more risk factors*	Baseline	Follow-up	P value	Baseline	Follow-up	P value
BMI ≥ 25 kg/m^2^	69.3	70.4	0.78	60.6	51.8	0.09
WHtR ≥ 0.5	92.9	94.1	0.60	24.4	17.7	0.12
WC ≥ 102 cm (males); ≥ 88 cm (females)	25.2	30.2	0.15	92.0	90.5	0.46
	**Females** (n = 532)					
	Sensitivity (%)			Specificity (%)		
*Two or more risk factors*	Baseline	Follow-up	P value	Baseline	Follow-up	P value
BMI ≥ 25 kg/m^2^	56.3	55.8	0.80	69.8	68.6	0.78
WHtR ≥ 0.5	87.5	86.7	0.80	34.4	30.0	0.29
WC ≥ 102 cm (males); ≥ 88 cm (females)	65.2	69.3	0.11	65.9	61.8	0.30

MetS, metabolic syndrome; BMI, body mass index; WHtR, waist-to-height ratio; WC, waist circumference

By contrast, enlarged WC occurred with a lower frequency in patients who developed MetS, particularly in males (sensitivity 30.2%) whereas in females, frequency was higher (sensitivity 69.3%), although below that of WHtR. Specificity was elevated in both genders (90.5 for males and 61.8 for females), indicating that the vast majority of MetS carriers have enlarged WC, and this occurs seldom in non-carriers. Finally, a BMI ≥ 25 kg/m^2^ had intermediate sensitivity and specificity regardless of gender (Table 3).

Of note, among to the 10.5% of subjects (55 males and 57 females) with a newly diagnosis of MetS at follow-up, WHtR ≥ 0.5 showed the highest sensitivity (96.3% and 91.1% for males and females, respectively) compared to BMI and WC.

**Fig 1:**
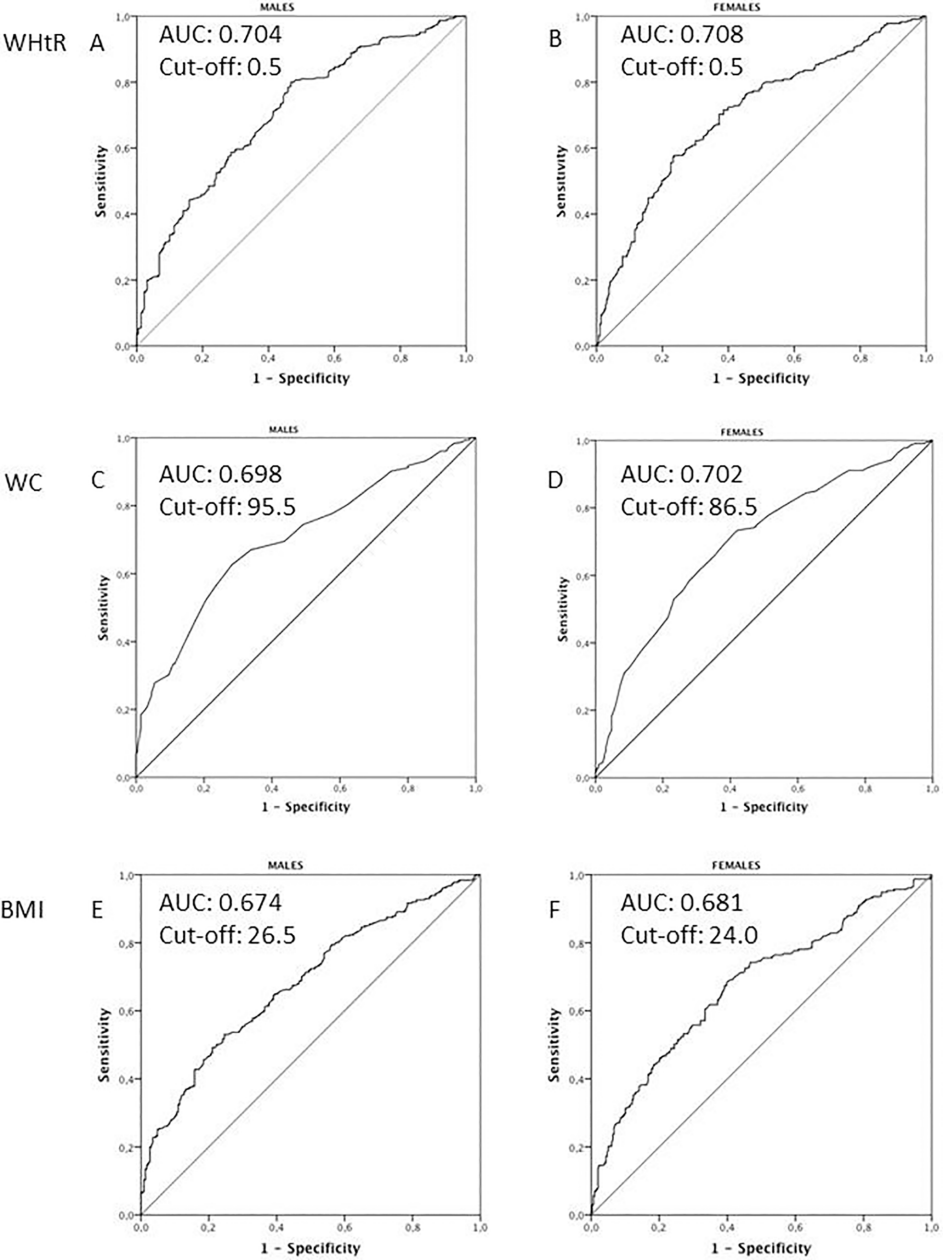
ROC curves for the different anthropometric indices. Cut-off values were assessed by the Youden index.

The ROC curves for a comparative assessment of sensitivity and specificity of the different anthropometric indices were, therefore, evaluated (Fig 1). Areas under the curve (AUCs) did not clearly indicate the best anthropometric index. Assessment of the best WHtR cut-off by the Youden index confirmed 0.5 as the best boundary for both genders. The maximum value of the Youden index may be used as a criterion for selecting the optimal cut-off. The Youden index is also defined as “informedness” or the probability of an informed decision (as opposed to a random guess). The Youden index also suggested an increase in the WC cut-offs compared to baseline (from 91.7 cm to 95.5 cm in males and from 84.7 cm to 86.5 cm in females) and it also indicated a slightly higher boundary value for BMI in both genders (from 24.7 to 26.5 kg/m^2^).

After excluding subjects with CV events at baseline, a sub-analysis was performed to test the association between anthropometric indices and CV events. Table 4 reports the odds ratios (ORs) for CV events: elevated WC had the highest (2.62, CI 1.18–5.78; p = 0.017), statistically significant, OR, followed by WHtR and BMI.

**Table 4:** Odds ratios (95% confidence interval (CI) for the association between cardiovascular events and three anthropometric indexes as criteria for MetS definition.

*Definition of MetS*:	Odds ratio	p value
BMI ≥ 25 kg/m^2^	2.11 (0.96–4.64)	0.650
WHtR ≥ 0.5	1.65 (0.75–3.63)	0.216
WC ≥ 102 cm (males); ≥ 88 cm (females)	2.62 (1.19–5.78)	0.017

MetS, metabolic syndrome; BMI, body mass index; WHtR, waist-to-height ratio; WC, waist circumferences.

## 4. Discussion

The evaluation of CV risk associated with MetS has become a standard goal in clinical practice. This goal includes the best choice of variables, both biochemical and body structure associated, allowing an optimal diagnosis of MetS, as well as providing a possible marker for CV risk prediction. Body variables associated with MetS have gained considerable interest, also in view of their simplicity of assessment and, in the case of WHtR, of the apparent uniformity of findings in different populations with marked differences in height [21]. In the present report, we evaluated, in a retrospective fashion, a series of over 1,000 patients from the same location and with an over 6-year follow-up with > 95% completeness of data [12].

A difference of 6–7 years between evaluations carries significant weight, since longitudinal analyses such as in the Uppsala County in Sweden, clearly indicated that a 5-year difference in age is associated with a significantly higher prevalence of MetS and, more so, of MetS components [22]. In addition, a long-term follow-up report found that the severity of MetS worsens with time [23]. In the present report, a considerable stability of the major biochemical and particularly body variables may be appreciated. The body variable reported at baseline as most frequent and most sensitive, *ie* WHtR with a breakpoint of ≥ 0.5, consistent with values in a large number of populations, maintained these qualifications.

The present study was also aimed at the re-evaluation of the predictive values of the three anthropometric indices assessed at baseline on the long-term development of MetS, as well as possibly, with CV events. Elevated WHtR was the index with the highest sensitivity, close to 100% for predicting the development of MetS; it had, however, a reduced specificity. Conversely, enlarged WC, particularly in males, had an elevated specificity, albeit with a lower sensitivity.

The ROC curves for the three body variables provided evidence of the validity of all three as predictive markers of MetS (Fig 1). While differences between these curves did not reach a statistical significance, the observed changes in these body variables, proved to be of definite clinical validity. In particular, it could be confirmed that WHtR has an excellent sensitivity, but a relatively low specificity, *ie* occurring frequently in non-MetS carriers. This finding may possibly explain the relatively low CV predictivity. Instead, WC, with a lower sensitivity but a high specificity, provided the highest ORs when tested in terms of prediction of CV events (Table 4).

This last conclusion contrasts, however, with data from the large meta-analysis by Ashwell *et al*. [21] on cardio-metabolic risk factors, involving more than 300,000 adults from several ethnic groups, assessing the discriminatory power of anthropometric indices in distinguishing adults with hypertension, type 2 diabetes, dyslipidemia, MetS and general CV outcomes. In this study, WHtR provided a significantly greater discriminatory power, compared to WC and BMI. In particular, WC improved discrimination for CV outcomes by 3% compared to BMI, whereas WHtR improved discrimination by 4–5% over BMI. In their study, by evaluating AUCs, WHtR proved to be a significantly better predictor compared to WC for diabetes, hypertension and all CV outcomes in both genders. In a prior investigation on this patient series, enlarged WHtR appeared to be associated to a raised progression of the cIMT, but the proposed association with a raised CV risk does not appear to be supported by the data of the present report [13].

The present study confirms WHtR as an excellent screening tool in identifying MetS carriers, but probably because of the lower specificity in our population, it did not appear to improve the prediction of CV outcomes. It should be noted that in the large UK meta-analysis [21] only 7 out of 32 considered studies provided data on AUC improvement (WHtR ≥ WC) and only for 6 populations data from both genders are reported. While the authors indicated that overall studies were consistent in showing a better association of CV outcomes with WHtR > WC > BMI, not all differences were statistically significant. WHtR was 9% better than BMI in predicting CV risk in males, but values for WC in females were not significantly better vs BMI. In men and women WHtR gave significantly better discrimination than WC for all outcomes, apart from the development of MetS, in which case WHtR did better only in women, possibly indicating that the considered populations differ from the one considered in the present report, where raised WHtR appears to be a most frequent finding in females [24]. Interestingly, however, in a follow-up of their study, Ashwell and Gibson indicated that a WHtR ≥ 0.5 may provide an early, simple and very predictive health risk indicator [25].

The present report thus provides clear evidence of a significant prevalence of MetS in middle-aged individuals from an Italian population. A limitation is that findings may not be transferable to other populations: a full generalizability of results from this large series may be, of course, the geographical location and the Caucasian ethnicity. In addition, life habits were not fully evaluated, *eg* physical activity, alcoholic beverages and the intake of drugs not reported in Table 1. The relatively low number of CV events could potentially limit the interpretability of the reported findings. The low CV risk is, however, in line with the recent observation of a substantially lower 5-year incidence of CV events (*eg*, 1.85% over 5 years in patient group with a predicted risk > 5%) [26], in a contemporary real-world non-diabetic population. Our findings may also support the very recent suggestion by Lee *et al*. [27] that body fat quantity and decreasing fat attenuation by computed tomography (CT), are linked to increased CV risk. In the present report, in fact increased abdominal fat, as witnessed by a raised WC, generally associated with fat attenuation and adipocyte hyperplasia [28], appeared to be the best anthropometric variable, predicting CV risk [29].
